# Safety of the effective radiation dose received during stroke hospitalization

**DOI:** 10.1590/1677-5449.210142

**Published:** 2022-01-07

**Authors:** Gregório Platero Canton, Gustavo José Luvizutto, Pedro Tadao Hamamoto, Marcos Ferreira Minicucci, Gabriel Pinheiro Modolo, André Petean Trindade, Rodrigo Bazan, Juli Thomaz de Souza

**Affiliations:** 1 Universidade Estadual Paulista – UNESP, São Paulo, SP, Brasil.; 2 Universidade Federal do Triângulo Mineiro – UFTM, Uberaba, MG, Brasil.

**Keywords:** stroke, radioprotection, radiation, CT scan, acidente vascular cerebral, radioproteção, tomografia computadorizada

## Abstract

**Background:**

Neuroimaging is widely used for diagnosis and treatment of stroke. However, little is known about whether the radiation doses received by patients comply with international safety guidelines.

**Objectives:**

The aim of this study was to evaluate the effective radiation dose received while in hospital for stroke and analyze its safety according to current guidelines.

**Methods:**

This cross-sectional study included 109 patients who were hospitalized and diagnosed with ischemic stroke. The National Institutes of Health Stroke Scale was used to evaluate stroke severity, the Bamford clinical classification was used for topography, and the TOAST classification was used for etiology. The computed tomography dose index and size-specific dose estimates were used to calculate the effective radiation dose (ERD) received while in hospital. A Mann-Whitney test was used to compare the ERD received by thrombolysed and non-thrombolysed patients. Non-parametric statistics were used to analyze the data with a 95% confidence interval.

**Results:**

During the study period, the median ERD received was 10.9 mSv. Length of stay was not associated with radiation exposure. No differences were demonstrated in ERD according to stroke etiology or Bamford clinical classification. Patients who had CT perfusion (only or in addition to CT or angiotomography) received the highest ERD (46.5 mSv) and the difference compared to those who did not (10.8 mSv) was statistically significant (p<0.001). No differences were found in the ERD between thrombolysed and non-thrombolysed patients. There was no correlation between ERD while in hospital and stroke severity.

**Conclusions:**

According to the current national guidelines, the protocol for examining images at our stroke unit is safe in terms of the ERD received by the patient while in hospital. There was no difference in the ERD received by patients stratified by thrombolytic treatment or stroke severity.

## Resumo

**Contexto:**

A neuroimagem é amplamente utilizada para o diagnóstico e tratamento do acidente vascular cerebral (AVC). No entanto, pouco se sabe se a dose de radiação recebida nesses exames está de acordo com as diretrizes internacionais de segurança.

**Objetivos:**

O objetivo deste estudo foi avaliar a dose de radiação efetiva (DRE) durante a hospitalização por AVC.

**Métodos:**

Trata-se de estudo transversal com 109 pacientes hospitalizados com diagnóstico de AVC isquêmico. A gravidade do AVC foi avaliada pela *National Institutes of Health Stroke Scale*, a topografia pela classificação clínica de Bamford e a etiologia pelo *Trial of ORG 10172 in Acute Stroke Treatment* (TOAST). O índice de dose recebida no exame de tomografia computadorizada (TC) e as estimativas de dose específicas foram usados ​​para calcular a DRE recebida durante a hospitalização. O teste de Mann-Whitney foi utilizado para comparar a DRE recebida por pacientes trombolisados ​​e não trombolisados. Estatísticas não paramétricas foram utilizadas para analisar os dados.

**Resultados:**

Durante o período do estudo, a DRE foi de 10,9 mSv. O tempo de internação não foi associado à exposição à radiação. Nenhuma diferença foi demonstrada na DRE de acordo com a etiologia e classificação clínica de Bamford. Os pacientes que fizeram perfusão (isolada, associada à TC ou angiotomografia) receberam a maior DRE (46,5 mSv) em comparação aos que não fizeram (10,8 mSv), sendo estatisticamente significativo (p <0,001). Não foram encontradas diferenças na DRE entre pacientes trombolisados e não trombolisados. Não houve correlação entre a DRE durante a hospitalização com a gravidade do AVC.

**Conclusões:**

De acordo com as atuais diretrizes nacionais, o protocolo de exame de imagens na unidade de AVC é seguro em relação à DRE recebido pelo paciente durante a internação. Não houve diferença na DRE dos pacientes de acordo com o tratamento trombolítico e a gravidade do AVC.

## INTRODUCTION

Stroke is a leading cause of death and functional disability worldwide and is an important public health problem. Approximately one in four people will have a stroke during their lifetime. It is a medical emergency, diagnosed using clinical and imaging tests, and computed tomography (CT) is the most commonly used test in the acute care of patients with stroke and, in some cases, magnetic resonance imaging.^1^^,^^2^

In patients with ischemic stroke, some procedures, such as intravenous thrombolysis or mechanical thrombectomy, can be performed in the acute phase with the aim of re-establishing cerebral reperfusion. Cerebral reperfusion treatments are extremely important in these cases; however, these treatments have some restricted indications and neuroimaging examinations are increasingly being used to improve indication of treatments in the acute phase as well as to monitor the patients’ clinical evolution.^2^^,^^3^ Regardless of the treatment received, the importance of correct diagnosis and definition of stroke type should be emphasized. Therefore, imaging examinations are essential in the care of these patients and the radiation dose received by the patient should comply with safety guidelines.

Radiation safety is widely studied in the field of physics to guarantee the safety of both occupationally exposed professionals and patients.^4^^,^^5^ The Food and Drug Administration (FDA) regulates radiation protection in the United States and in Brazil the National Nuclear Energy Commission (CNEN) has established an annual dose limit of <100 millisieverts (mSv) to prevent serious damage to health, such as acute radiation syndrome.^6^ The effects of excess radiation received by humans are significant as it can cause cell degradation and formation of other molecular structures. Smaller doses result in nausea, vomiting, changes to blood counts, and bleeding. Higher doses can lead to neurological effects and death. Radiation exposure can also increase the likelihood of cancer.^7^

Previous studies have shown that the radiation dose administered during acute stroke care is safe.^8^^,^^9^ Considering the life expectancy of the population, high recurrence of stroke, increasing occurrence of stroke in young individuals, and development of new imaging techniques and stroke treatments, the radiation doses received while in hospital for stroke must be checked to ensure radiological protection of patients. Therefore, this study aimed to evaluate the effective radiation dose (ERD) received while in hospital for stroke and analyze its safety according to current national guidelines. Additionally, this study also aimed to determine differences in the ERD received during different types of neuroimaging examinations and treatments administered during the acute phase and identify any relationships between the ERD received and stroke severity.

## METHODS

### Design and population

This cross-sectional observational study included 109 patients aged ≥18 years diagnosed with ischemic stroke based on clinical evaluation and neuroimaging findings, who were hospitalized at the Stroke unit at the Botucatu Medical School between March and August 2019. Patients with other neurological diagnoses and those with insufficient data in their electronic medical records were excluded. The study was approved by the ethics committee (Number: 3.199.087) and followed the tenets of the Declaration of Helsinki.

### Sample size calculation

According to an a priori sample size calculation, considering statistical significance of 0.05 (95%), effect size of 0.30, and the test power (type II error) of 0.90, a total sample of 88 participants was needed. This sample size was calculated using the G*Power 3.1 application.

### Data collection

Data were collected related to the clinical and neurological evaluations performed by the medical team, including those from anamnesis, on history of diseases, presence of thrombolysis, age, sex, length of hospital stay, previous disabilities (assessed using the modified Rankin Scale [mRs]),^10^ stroke severity (evaluated using the NIHSS),^10^ stroke classification (based on underlying pathophysiology and clinical presentation using the Bamford clinical classification),^11^ Trial of ORG 10172 in Acute Stroke Treatment (TOAST) classification of acute ischemic stroke,^12^ and the computed tomography dose index (CTDI) of the imaging examinations performed while in hospital.^13^^-^15 The size-specific dose estimate (SSDE) was calculated using CTDI values. The SSDE is defined as a patient dose estimate that considers corrections based on the size of the patient using linear dimensions measured on the patient or the patient’s images.^13^^-^15

### Stroke unit imaging exam protocol

During hospitalization, the following imaging examinations were performed: brain CT without contrast, neck and brain angiotomography, and multislice computed tomography (MSCT). The protocol for imaging examinations for patients in the acute phase of stroke consists of brain CT, angiotomography, or MSCT at admission and control re-examination within 48 h. For patients who are admitted after the acute phase of stroke, only one imaging examination is performed to confirm the diagnosis. If symptoms worsen or a new symptom develops while in hospital, another imaging examination is requested.^2^^,^^13^

### Brain computed tomography

Non-contrast head CT allows fast and safe evaluation to rule out hemorrhage or space-occupying lesions.^9^ Studies were performed on a Toshiba Action 16 multislice scanner in the helical mode; 200-1200 images were acquired in each examination.

### Multislice computed tomography

The use of MSCT as an imaging method using cerebral perfusion tomography enables dynamic visualization of lesions and obstructions owing to its accuracy and quality of image acquisition. Images were acquired using helical, axial, and cine sections.^14^

### Brain angiotomography

Angiotomography enables observation of the structures seen on CT and vessels, veins, and arteries due to the reaction of the iodinated contrast with the X-rays. Images were acquired using helical and axial sections in the sequence of helical, axial, and helical acquisition, where the axial section was the cross-section of the skull.^15^

## NEUROLOGICAL ASSESSMENT

For clinical and neurological evaluations, stroke care scales were used on admission and while in hospital.

### National Institutes of Health Stroke Scale

The NIHSS score was used to assess stroke prognosis and severity. This comprises 11 items related to neurological tests to verify the effects of brain injury on the level of consciousness, loss of visual fields, facial paralysis, motor deficits in the upper and lower limbs, limb ataxia, language deficits, and unilateral inattention. Scores range from 0 to 42, with higher scores indicating greater stroke severity.^10^

### Bamford clinical classification

The Bamford clinical classification categorizes patients with ischemic stroke into four categories according to their symptoms and signs, enabling the probable pathology of the stroke to be understood and assisting in treatment and prognosis. The categories are lacunar stroke (LACS), partial anterior circulation stroke (PACS), posterior circulation syndrome (POCS), and total anterior circulation stroke (TACS).^11^

### Trial of ORG 10172 in Acute Stroke Treatment (TOAST) classification

The TOAST classification categorizes stroke into five stroke subtypes according to etiology. The subtypes include large-artery atherosclerosis, cardioembolism, small-vessel occlusion, stroke of other determined etiology, and stroke of undetermined etiology.^12^

## EVALUATION OF RADIOLOGICAL PROTECTION

In Brazil, the CNEN is responsible for regulating, licensing, and supervising production and use of nuclear energy. In this context, resolution 164/14 from March 2014 sets out the CNEN 3.01 standard establishing radiation protection guidelines that define the basic requirements of radiation protection to protect patients from exposure to ionizing radiation. The standard limits the effective dose to 100 mSv. This is a cumulative dose calculated over a 1-year period, although in this study we only used the dose received when hospitalized in the Stroke Unit.

## CALCULATION OF THE EFFECTIVE DOSE

The ERD received by patients while in hospital for stroke was calculated using the formula E = ∑ Wt. HT, where Wt corresponds to the correction factor for the weight of each tissue, for which values of 0.01, 0.1, and 0.05 were used for skin and brain, bones, and thyroid, respectively, and where HT corresponds to the SSDE value. The SSDE values in this study were calculated using the image closest to the starting point of the tomography measurement of the anteroposterior (AP) thickness of each patient, disregarding the height of the table, thus correcting for effective size using the equation: -3.7448E0 + 1,6717E0X-1,33895E-2X.^2^

## STATISTICAL ANALYSIS

The distribution of data was assessed using the Shapiro-Wilk test. The variables examined did not follow a normal distribution and non-parametric statistics were used to analyze the data with a 95% confidence interval. Variables are expressed as mean ± standard deviation or medians and 25th and 75th percentiles. Examination types were compared using the Kruskal-Wallis test, followed by Dunn’s test for non-parametric data. The Mann-Whitney test was used to verify the median ERD received during different neuroimaging examinations (perfusion CT and non-perfusion CT) and according to treatment received in the acute phase (thrombolysed and non-thrombolysed patients). The Kruskal-Wallis test was used to verify the ERD received according to the TOAST and Bamford clinical classifications, and Spearman’s rank correlation coefficient was used to verify the relationship between the ERD received and stroke severity. The significance level was set at 5%. Sigma Plot 12.0 (Dundas Software LLC, Germany) was used to perform all statistical analyses.

## RESULTS

**Figure 1:**
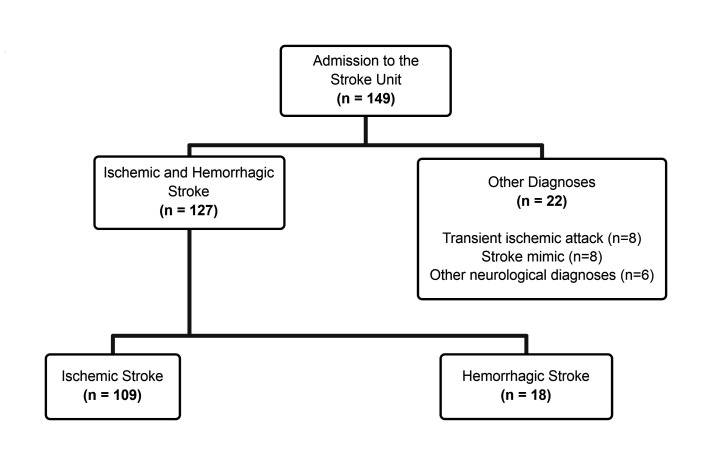
Flowchart of patients included in the study.

During the study period, 149 patients were admitted to the stroke unit. Of these patients, 109 were included in the study. A flowchart of patient recruitment and inclusion is presented in Figure 1. The average length of hospital stay was 7 days, 13.8% of patients were thrombolysed, and the average radiation received was 10.9 mSv. Length of stay was not correlated with radiation exposure (r = 0.213; p = 0.456). The general characteristics of the study patients are summarized in Table 1.

**Table 1:** General characteristics of patients admitted to the stroke unit with ischemic stroke (n=109).

**Variables**	
Male sex, N (%)	64 (58.7)
Age (years)	68.4±14.2
Previous stroke/TIA, N (%)	20 (18.3)
Previous AMI, N (%)	7 (6.4)
Hypertension, N (%)	77 (70.6)
Diabetes mellitus, N (%)	28 (25.7)
Dyslipidemia, N (%)	14 (12.8)
Smoking, N (%)	32 (29.4)
Alcoholism, N (%)	16 (14.7)
Arrhythmia, N (%)	11 (10.1)
Length of stay (days)	7 (4.5 – 9)
Thrombolysis, N (%)	15 (13.8)
Death, N (%)	6 (5.5)
ERD (mSv)	10.9 (7.2 – 22.1)
NIHSS at admission	5 (2 – 9.5)
NIHSS at discharge	2 (1 – 6.5)
Previous mRs	0 (0 – 1)
mRs at discharge	2 (1 – 4)

TIA: transient ischemic attack; AMI: acute myocardial infarction; mSv: millisievert; NIHSS: National Institutes of Health Stroke Scale; mRS: modified Rankin Scale.

**Figure 2:**
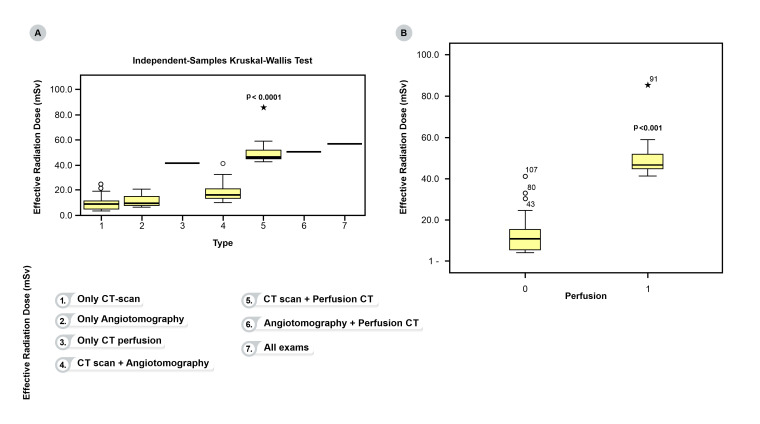
(A) ERD in all neuroimaging examination types; (B) Comparison of ERD between patients who had CT perfusion (isolated or associated with CT or angiotomography) with those who did not.

All patients underwent a minimum of one imaging examination according to the requirements assessed in each specific case. The distribution of the tests performed at the stroke unit is shown in Figure 2. According to the Bamford clinical classification, 45 patients (38.5%) had LACS ischemic stroke. According to the TOAST classification, 59 (54.1%) patients presented with stroke of undetermined etiology. No differences in ERD were identified between Bamford clinical classifications (p = 0.65) or between stroke etiologies (p = 0.58). These results are presented in Table 2.

**Table 2:** Median and confidence interval (CI) of ERD received according to the Bamford clinical classification and TOAST classification of patients admitted for ischemic stroke (n=109).

	**Effective dose (mSv)**	**p**
Bamford classification		
LACS (n=42)	10.8 (5.5-17.2)	
PACS (n=20)	14.1 (7.8-21.2)	0.65
POCS (n=28)	12.5 (5.7-42.5)	
TACS (n=19)	12.7 (10.4-45.6)	
TOAST classification		
Cardioembolism (n=20)	10.8 (5.5-22.3)	
Large-artery atherosclerosis (n=16)	13.0 (9.5-27.9)	
Undetermined etiology (n=59)	10.9 (6.4-21.9)	0.58
Other determined etiology (n=3)	10.9 (9.3-58.9)	
Small-vessel occlusion (n=11)	14.9 (10.9-41.2)	

mSv: Millisievert; LACS: lacunar stroke; PACS: partial anterior circulation stroke; POCS: posterior circulation syndrome; TACS: total anterior circulation stroke; TOAST classification: Trial of ORG 10172 in Acute Stroke Treatment (TOAST) classification. The Kruskal-Wallis test was performed.

Figure 2A illustrates the ERD for all neuroimaging examination types: patients who had only a CT scan (8.7 mSv); only angiotomography (9.3 mSv); only CT perfusion (41.4 mSv); CT scan plus angiotomography (16.394 mSv); or CT scan plus CT perfusion (46.5 mSv). Only one patient had angiotomography plus CT perfusion. Patients who had CT perfusion (isolated or associated with CT or angiotomography) received the highest effective doses (46.5 mSv) and the difference compared to those who did not (10.8 mSv) was statistically significant (p<0.001) (Figure 2B).

**Figure 3:**
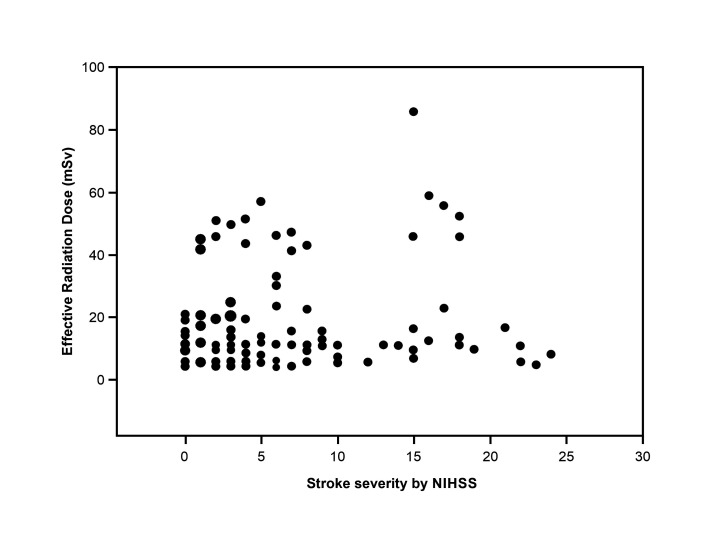
Correlation between the ERD while in hospital and stroke severity.

There were no differences in ERD (p = 0.99) between thrombolysed (10.9 [6.8–23.4] mSv) and non-thrombolysed (11.3 [7.3–22.0] mSv) patients with ischemic stroke. There was no correlation between the ERD received by patients with ischemic stroke admitted to the stroke unit and stroke severity according to the NIHSS score (r = 0.045; p = 0.641). Figure 3 illustrates the absence of correlation between the ERD and stroke severity.

## DISCUSSION

This study primarily aimed to determine the amount of X-ray irradiation received while in hospital for stroke and analyze its safety according to current national guidelines. Additionally, we analyzed the differences in the amount of irradiation received by thrombolysed and non-thrombolysed patients and the relationship between the amount of irradiation received and the severity of the stroke. The protocol for performing imaging tests employed in the stroke unit was safe in terms of the ERD received by patients while in hospital and no differences were observed in the effective dose according to treatment, severity, etiology, or clinical signs of stroke.

Monitoring radiation exposure at all stroke centers as quality control is a practical tool for detecting sources of systemically high radiation exposure.^16^ Radiological studies are increasingly being extended to select patients who will benefit from current therapies for treatment of acute stroke. Most centers in the world are currently using tomography instead of magnetic resonance, with supplementary arterial studies using angio-CT (CTA) and in some centers with CT perfusion studies (CTP). Performing supplementary CT techniques such as CTA and CTP for assessment of acute stroke increases the total radiation exposure.

Use of advanced imaging tests, such as CT, CT angiography, and MSCT, allows the care team to promptly make the most accurate diagnosis, and can assist in decision-making regarding the most effective cerebral reperfusion treatment. Therefore, imaging tests are required for stroke diagnosis. Supplementary CT techniques (CT angiography and CT perfusion) for assessment of acute stroke increase total radiation exposure.^9^ With the developments in technology over recent years, tests have become increasingly safer in terms of the amount of radiation required to obtain good-quality images.^17^

A previous study confirmed that the radiation emitted by cranial CT devices in other clinical situations is safe regardless of the protocol adopted.^18^ In our study, the stroke unit care protocol was safe in terms of the radiation received, regardless of the type of treatment. Mnyusiwalla et al.^8^ showed that a comprehensive CT acute stroke protocol delivered a mean effective dose of 16.4 mSv, which is approximately six times the dose of an unenhanced CT head scan. Zensen et al.^9^ reported that the median dose in patients was 8.3 mSv, 5.5. mSv of which were delivered for the CT perfusion component. According to the Brazilian health surveillance agency, which regulates radiation exposure in the country, a dose of 100 mSv is considered safe for voluntary exposure. In our study, the average radiation received was 10.9 mSv. This limit is below any deterministic limit; thus, the protocol used in the stroke unit is deemed safe.

Our results demonstrated that the radiation received by patients while in hospital for stroke did not differ according to the etiology of stroke assessed using the TOAST classification. A previous study including patients in the acute phase of stroke showed that the characteristics of the thrombus in cardioembolic stroke are different from those in non-cardioembolic stroke, suggesting use of CT as a diagnostic tool to evaluate the cause of stroke in clinical practice.^19^ However, further studies comparing the amount of radiation received according to the cause of stroke are needed.

An ischemic event may lead to a transient, potentially reversible, condition known as ischemic penumbra, in which reduced blood flow in an area of the brain can be reversed before injury with prompt and appropriate treatment. In these cases, revascularization therapy removes the thrombus by promoting recanalization of the cerebral vessel and restoration of blood flow, resulting in tissue recovery. If cerebral reperfusion does not occur promptly, the lesion in the affected region will progress to a permanent neurological deficit.^20^

To avoid this complication, the speed of service is essential and imaging tests are important for choosing the appropriate reperfusion treatment. The most commonly used reperfusion treatments in clinical practice are intravenous thrombolysis and mechanical thrombectomy. Thrombectomy was not performed in any of the patients in our study, while 15 patients received intravenous thrombolysis. No differences were observed in the amount of radiation received by thrombolysed and non-thrombolysed patients. Thrombolysed patients underwent the same imaging tests as the other patients, resulting in a similar ERD.

One limitation of our study is the small percentage of patients who received intravenous thrombolytics (n=15) and there was also a large number of patients with undetermined etiology in the TOAST classification. No mechanical thrombectomy was conducted in this study and this technique may increase radiation exposure while in hospital. Patient care after a stroke is interdisciplinary, complex, and urgent. The effective radiation dose received by patients under these circumstances reflects the “real world” circumstances of a stroke unit. Imaging examinations are important tools for diagnosis and decision-making to choose the most appropriate treatment in each case.

## CONCLUSION

According to the current national guidelines, the protocol for examining images at our stroke unit is safe in terms of the ERD received by the patient while in hospital. There was no difference in the ERD received by patients grouped according to thrombolytic treatment or stroke severity.
